# Changes in the analysis of temporal community dynamics data: a 29-year literature review

**DOI:** 10.7717/peerj.11250

**Published:** 2021-04-08

**Authors:** Hannah L. Buckley, Nicola J. Day, Gavin Lear, Bradley S. Case

**Affiliations:** 1 School of Science, Auckland University of Technology, Auckland, New Zealand; 2 School of Biological Sciences, Victoria University of Wellington, Wellington, New Zealand; 3 School of Biological Sciences, University of Auckland, Auckland, New Zealand

**Keywords:** Biological communities, Multivariate analysis, Spatiotemporal change, Community ecology, Community dynamics, Time series, Descriptive analysis, Quantitative analysis

## Abstract

**Background:**

Understanding how biological communities change over time is of increasing importance as Earth moves into the Anthropocene. A wide variety of methods are used for multivariate community analysis and are variously applied to research that aims to characterise temporal dynamics in community composition. Understanding these methods and how they are applied is useful for determining best practice in community ecology.

**Methodology:**

We reviewed the ecological literature from 1990 to 2018 that used multivariate methods to address questions of temporal community dynamics. For each paper that fulfilled our search criteria, we recorded the types of multivariate analysis used to characterise temporal community dynamics in addition to the research aim, habitat type, location, taxon and the experimental design.

**Results:**

Most studies had relatively few temporal replicates; the median number was seven time points. Nearly 70% of studies applied more than one analysis method; descriptive methods such as bar graphs and ordination were the most commonly applied methods. Surprisingly, the types of analyses used were only related to the number of temporal replicates, but not to research aim or any other aspects of experimental design such as taxon, or habitat or year of study.

**Conclusions:**

This review reveals that most studies interested in understanding community dynamics use relatively short time series meaning that several, more sophisticated, temporal analyses are not widely applicable. However, newer methods using multivariate dissimilarities are growing in popularity and many can be applied to time series of any length.

## Introduction

A hallmark of the Anthropocene Epoch is the increasing pace and variance of environmental change (Lewis & Maslin, 2015; Dornelas et al., 2019; Eriksson & Hillebrand, 2019). Thus, more than ever, there is need for ecologists to monitor, quantify, analyse and predict temporal changes in the structure of biological communities as a function of ongoing changes in climate, land use, and other biotic and abiotic drivers. Temporal datasets from repeated measures studies such as long-term ecological research network sites (Soltwedel et al., 2016), marine sea catch (Royer & Fromentin, 2006) and microbial soil datasets (Storkey et al., 2016) are becoming longer and more commonly used for understanding community dynamics. Combined with significant increases in computational speed and capacity over this time, the diversity of methods available to interrogate these data has also increased. Many analysis methods used to date are poorly suited to the analysis of long and intensive time series data, yet a suite of recent analysis methodologies have significant potential to provide valuable new insights into key trends that might otherwise be ignored or misinterpreted from the analysis of long-term temporal datasets (e.g., Tonkin et al., 2017; Avolio et al., 2019; De Cáceres et al., 2019; Legendre, 2019). Given these potential shifts in available analysis methods, datasets, and computing capabilities associated with the study of temporal community dynamics, we were interested in exploring if and how research in this field has changed in response to these putative developments over the past three decades.

Simple community measurements, such as the number of taxa (‘species richness’), are frequently used to monitor and predict change in biodiversity; these measures, however, offer no insight into more complex community changes, such as in the relative commonness and rarity of the species present (Hillebrand et al., 2018; Blowes et al., 2019). For example, a single invasive species could have devastating effects on relative abundances of all other taxa, but little impact on univariate measures such as biomass or species richness (Bradley et al., 2019). Further, there are often nonlinear and/or lag effects of disturbances or human-caused changes on communities that can only be fully disentangled by considering a more comprehensive range of community measures through time (Magnuson, 1990, Lindenmayer et al., 2012). Thus, for our review we focus specifically on studies that have characterised changes in multivariate community composition: the shifts in taxonomic identities, and potentially relative abundances over time. Most often these data are collated as a site (sample) by species (taxon) matrix.

Knowledge of the types of multivariate analyses being applied in the temporal community dynamics literature is crucial because this is a constantly evolving field at the challenging interface of biology, statistics and bioinformatics (Buckley et al., 2021). A first step towards choosing effective methods to apply to a given dataset is an awareness of how available methods are currently being applied across different types of temporal community datasets, collected to address particular research questions, such as testing for seasonal dynamics (Tammert et al., 2015) or climate impacts (Magalhaes et al., 2007), often using a variety of data types (e.g., the relative abundance of bacterial DNA sequences (Buscardo et al., 2018)), or the number of functional groups of fishes (Wilson et al., 2008). Breaking down discipline barriers by using comparable analysis methods on different kinds of datasets will advance our understanding of shifts in biological communities globally (e.g., Shackelford et al., 2017; Collins et al., 2018) and assist in the choice of analysis method, which is often complicated for both biological and statistical reasons.

In this paper, we take a cross-disciplinary approach to review empirical studies published up until the start of 2019 that have conducted analyses of multivariate temporal community datasets to gain insight into how researchers have applied analysis methods over the past several decades. We summarise trends in the use of multivariate methods to analyse compositional community temporal dynamics and provide a summary of the available methods. Specifically, we asked: (1) have different taxa, habitat types or spatiotemporal scales been investigated more or less comprehensively over the past three decades; (2) which analysis methods are applied most often, and has this changed over time; (3) does the level of temporal replication affect the analyses applied; and (4) are analyses applied differentially across research questions? The answers to these questions will assist us to identify both the methods used in temporal community analysis and gaps in our understanding of community change using data collected from diverse combinations of spatiotemporal scales, taxa and habitats.

## Survey Methodology

We examined the literature for studies investigating changes in ecological communities over time. Specifically, we searched the ISI Web of Science Core Collection for articles published until 31 December 2018 using the following search statement: (“temporal” AND “composition*” AND “communit*” AND (“community dynamic*” OR “temporal dynamic*” OR “temporal community varia*” OR “community change” OR “compositional change”)). We refined this search to include only document types listed as ‘articles’ and published in ‘English’. This search yielded 820 articles for review. Of these, we excluded 257 papers that lacked community data (i.e. those that included fewer than three taxa), had no temporal replication or pooled all temporal data, used only artificial communities or simulated datasets, or used space-for-time substitution (e.g., using chronosequences). Although we constrained the search to the end of 2018, we did not constrain the search window for the beginning date. However, our search terms only returned papers after 1990, reflecting that the specific terms we used were not part of the compositional change literature prior to the 1990s. We also note that this also excluded a great deal of the literature on ecological succession, which uses more specific terminology.

We extracted and collated key attributes relating to each study in the remaining 539 papers from the ISI database (e.g., publication year, number of citations). Specific study attributes were determined from the body text of each manuscript, including information on (i) habitat type (e.g., estuarine); (ii) continent (e.g., South America); (iii) taxonomic identity (e.g., vertebrate); (iv) manipulation (e.g., observational study, experimental manipulation); (v) spatial extent (e.g., local, global); (vi) temporal grain and extent (e.g., minutes, months); and (vii) the key perceived research aim (e.g., exploration of yearly dynamics). We categorised organisms being studied into the following taxonomic groups: plants (Kingdom Plantae); vertebrates (Phylum Chordata); invertebrates (Phyla Annelida, Arthropoda, Cnidaria, Ctenophora, Echinodermata, Mollusca, Platyhelminthes, or Porifera); microeukaryotes (algae and Kingdoms Fungi and Protista); prokaryotes (Domains Bacteria and Archaea); and viruses (non-cellular pathogenic organisms). Spatial extent was determined relative to the dispersal ability of the taxa involved, e.g., a ‘local’ study would be across a larger area for birds than for soil invertebrates. The categories were (1) small scale: micro, point, and local scales (within-populations) and (2) large scale: regional (among sites), continental and global scales. Research aims were classified broadly using statements in the abstract and introduction regarding the authors intentions. If hypotheses were tested, these were used to classify the study aims as relating to environmental interactions, taxon interactions or spatiotemporal variation at different scales.

We recorded the types of multivariate analysis used in each study, that is, analyses that use the taxon-sample matrix as an input (Table 1). The search criteria resulted in papers that were based on a wide variety of data types including presence-absence and relative abundances; however, we found that data type did not influence the type of analysis performed and so is not discussed further. Similar analysis methods were grouped together, for example, ‘Descriptive Methods’ were all those that showed simple, visual presentation of data such as Venn diagrams showing overlap in taxonomic composition as well as tables and graphs of relative abundances over time. Two methods were included in this review because they analysed aggregate community data, but were not technically multivariate, i.e., using the taxon-sample matrix as the input: temporal stability (*a.k.a*. coefficient of variation) and multiplicative change. Multi-taxon papers were analysed separately as independent data points if the taxa were sampled and analysed independently. The full list of papers reviewed and collated review data are provided in the Data S1.

**Table 1:** Brief description of methods for the temporal analysis of multivariate community data encountered in this review, the number of uses out of a total 1,261 analyses recorded, and the key references illustrating and/or explaining each method or set of method. Note that dissimilarity refers to pairwise similarity or dissimilarity measures calculated between pairs of samples, such as Euclidean distance or Bray-Curtis dissimilarity (for a full explanation and summary of these methods, see Legendre & Legendre, 2012).

Analysis method or set of methods	Number of uses	Description	Key references
Descriptive methods	425	Simple, mostly visual, representations of compositional change such as bar graphs, line graphs, heat maps, and tables showing change in relative abundances. This set of methods also includes Venn diagrams and simple lists of species showing comparisons of composition from samples taken at different times.	Vallès et al. (2014), O’Sullivan et al. (2015), Pereira et al. (2017)
Ordination	315	Ordination is a set of dissimilarity-based methods that summarise multivariate community data by optimising relationships between high-dimensional samples and taxa in low-dimensional space to detect the dominant ecological gradients in communities. When samples are taken at different times, these can be compared by labelling points by time in various ways, e.g., trajectories, symbols, envelopes.	Legendre & Legendre (2012), Anderson & Willis (2003), Day & Buckley (2013), Auber et al. (2017), Palmer (2000)
Raw dissimilarity	257	Raw dissimilarity values are used in a wide variety of methods from simple, e.g., mean dissimilarity values for temporal data subsets used in subsequent graphs, analyses or maps, to more complex methods that decompose beta diversity values into components representing the degree of nestedness and turnover among temporal samples. Raw dissimilarity values can be calculated in two main ways: (1) between temporal samples as a measure of compositional change and (2) between spatial samples at different times, which are then averaged and subsequently compared. Both approaches are included within this group of methods. Note that ordination, clustering, and time-lag analyses are also based on dissimilarity values.	Anderson et al. (2011), Bashan et al. (2016), Collins (1992), Collins (1992), Barros et al. (2014), Baselga & Orme (2012), Legendre & De Cáceres (2013), Lamy et al. (2015)
Time-lag analysis	113	Time-lag analyses involve relating the amount of compositional change to the amount of change in time across increasing temporal distances, called ‘lags’. There are two ways of assessing the time-lag effect on community dissimilarity: graphically (‘time-decay curve’) and statistically (time-lag regression analysis).	Collins & Xia (2015), Kampichler et al. (2012), Lightfoot et al. (2012), Dimitriu et al. (2013)
Cluster analysis	82	Cluster analysis is a catch-all term applied to dissimilarity-based methods that either group samples together (agglomerative methods) or split all samples into sub-groups (divisive methods); the clustering is based on the dissimiliarities between groups of samples (Legendre & Legendre, 2012). There are two approaches for using cluster analysis to assess community dynamics: (1) detecting transitions of sites from one cluster to another over time, and (2) comparison of compositional differences among clusters of repeated observations. In the first approach, a separate cluster analysis is computed for each temporal sample of community data. In the second approach, all samples taken at different times are included in a single cluster analysis.	Day & Buckley (2013), Araujo et al. (2008)
Turnover rates	28	The degree of temporal variation in community composition can be assessed by calculating the turnover rate of the community (a.k.a. ‘temporal turnover’ or ‘species turnover’), which is a measure of the rate of change in taxonomic composition for a ‘site’ over time. Turnover rates are calculated in a variety of ways using combinations of colonisation, immigration, extinction, mortality, recruitment, and survival, and can be as simple as the percent change in species composition between time points.	Diamond (1969), Aguirre et al. (2003)
Network analysis	13	Ecological network analysis methods recognise that an ecological community is a complex biological system comprised of interconnected units whose associations can be modelled mathematically using constructs such as vertices (representing taxa) and edges, which are the connections between the vertices (representing ecological interactions). In the context of investigating community dynamics, patterns of species’ associations and community memberships are most typically visualised as a topological network diagram created from taxon abundance data collected at multiple time points. From the network topology, a variety of statistics can be calculated that measure variability in community composition and interactions at particular times, which can then be compared.	Blonder et al. (2012), Newman (2010)
Temporal stability (a.k.a. coefficient of variation)	9	The coefficient of variation (CV) is used to measure temporal stability of the abundance or biomass of an individual taxon, or group of taxa, across all times (not space). The CV is the ratio of the standard deviation to the mean, and often is multiplied by 100 to obtain a percentage. The mean of the CV values for all taxa (e.g., abundance or biomass) is used as an aggregate measure of temporal stability for a whole community; smaller values imply greater stability. Such values are often presented in tables as a measure of variation or are sometimes used as a response variable against other variables of interest.	Grossman, Dowd & Crawford (1990), Brown & Lawson (2010), Hector et al. (2010), Doxa et al. (2012)
Machine learning methods	8	Machine learning is branch of computer science that deals with the development of learning algorithms that are used to explore large, multivariate datasets, and has the primary aim of generating accurate, predictive models (Carbonell, Michalski & Mitchell, 1983). These methods are very flexible and can take multivariate input datasets with large numbers of predictor variables, including space and time, allowing simultaneous investigation of spatial and temporal community dynamics.	Vallès et al. (2014), Sheaves, Johnston & Connolly (2010), De’Ath (2007)
Moran Eigenvector Maps (MEMs)	4	Analyses using Moran Eigenvector Maps (also referred to as principal coordinates of neighbour matrices; PCNM) result in a matrix of uncorrelated temporal variables that characterize scales of temporal variation in composition that can be used in another multivariate analysis such as an ordination, e.g., distance-based redundancy analysis. If the ordination is paired with variance partitioning, a Venn diagram showing the amount of variance explained by eigenvectors representing different scales of temporal variation in composition can be generated.	Legendre & Gauthier (2014)
Synchrony	1	Synchrony is calculated as a single value representing the degree to which taxa are changing in a similar way over time. It is generated for a given sample by taxon matrix across a set of times. It can be compared for different time windows or different subsets of samples, e.g., experimental treatments.	Loreau & De Mazancourt (2008), Gouhier & Guichard (2014)
Nestedness analysis	1	Nestedness analysis (*sensu* Patterson & Atmar, 1986) is usually applied to spatial data and the analysis can be repeated for different time points; however, it can be applied to samples taken across times to look at whether sites are becoming more or less nested over time. For instance, if younger sites were nested within older sites, this may indicate that sites were becoming more homogeneous over time.	Collins et al. (2017)
Multivariate regression modelling	1	There are a range of regression approaches that simultaneously model individual species, such as ‘multispecies N mixture models’ and ‘joint species distribution models’. These methods can be used to model explicit, quantitative hypotheses of community change, with a focus on interactions among taxa. Some methods allow for useful additions, such as accounting for uncertainty in the detection of taxa. However, due to their complexity and high computational requirements, most of these approaches have yet to be implemented for datasets with large numbers of species. Multivariate regression models quantify the relative effects of species (or species groups) interactions, environmental covariates, spatial structure, and observation error on relative abundances through time.	Hampton et al. (2013), Dorazio, Connor & Askins (2015), Ovaskainen et al. (2017a)
Multiplicative change	1	The change in percent cover of a taxon, or group of taxa, at a single site can be used to obtain a ‘growth rate’ using a linear regression equation. This rate of change can then be used in a regression or ANOVA to assess predictors of change for each taxon or group (‘multiplicative change’). For example, different sites or sets of sites, can be compared by their relative change in the percent cover of different functional groups, as long as communities consist of taxa with similar ecology and life-histories, e.g., grassland plant communities in different moisture regimes.	Debinski et al. (2010), Collins & Xia, 2015
Compositional pivot days	1	Compositional pivot days is a dissimilarity-based method developed by Lellouch et al. (2014). It is appropriate for situations where we want to test for the occurrence of rapid changes in taxonomic composition across one point in time, such as the application of an experimental treatment. It works by comparing pairwise distance values for pairs of temporal observations taken at the same sample location to identify the time points where rapid shifts in composition occurred. The method generates a list of time points (‘pivot days’) at which significant shifts in composition have occurred.	Lellouch et al. (2014)

## Results

**Figure 1:**
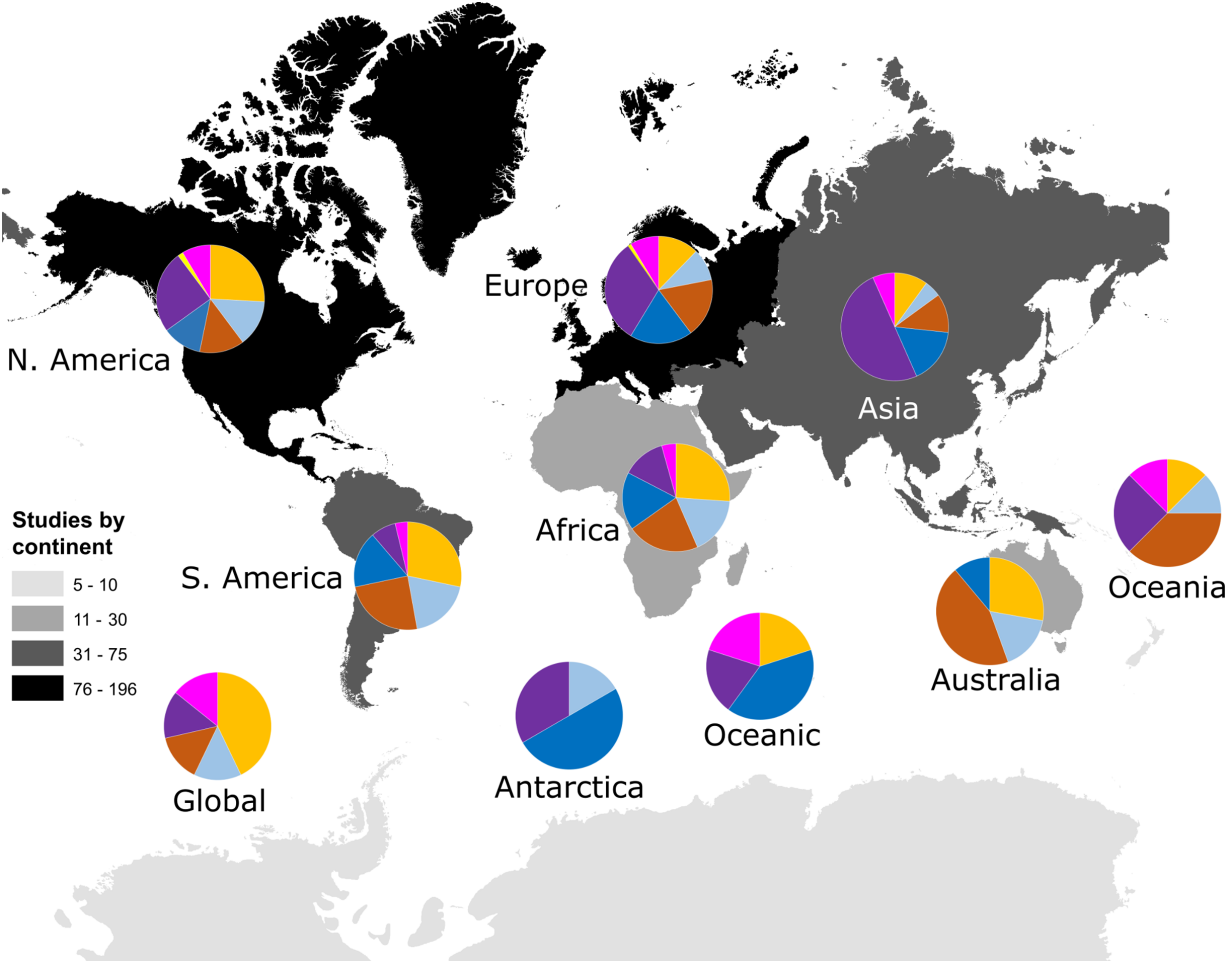
Number of studies identified by our search criteria from each continent. Numbers of studies were: Global (*n* = 7), Oceanic (*n* = 5), Africa (*n* = 23), Antarctica (*n* = 6), Asia (*n* = 60), Australia (*n* = 18), Europe (*n*= 196), North America (*n* = 186), Oceania (*n* = 8) and South America (*n* = 53). Darker colours indicate more studies have been conducted within those continents, including Oceania. Pie charts show the taxonomic focus of study data from each continent as being on plants (orange), vertebrate animals (light blue), invertebrate animals (brown), microeukaryotes (blue), prokaryotes (purple), viruses (yellow), or mixed taxonomic groups (pink).

Our search identified 521 research papers investigating multivariate community dynamics from 1990 to 2018 and a total of 1,259 analyses. Few publications (22 papers; 4%) contained more than one independent dataset; those instances were treated as multiple studies that were analysed as separate data points (increasing our total sample size *n* to 548). The majority of studies (68%) were conducted within North America and Europe (Fig. 1). The most commonly studied taxon was prokaryotes (28%), followed by plants (19%), invertebrates (17%), microeukaryotes (16%), vertebrates (12%) and viruses (1%); studies considering multiple taxa, such as marine microeukaryotes and invertebrates, comprised 7% of studies (Fig. 1). Studies in the southern hemisphere were more likely to be focussed on the analysis of plant or invertebrate data, whilst analyses of prokaryote community data were more common in the northern hemisphere (Fig. 1).

**Figure 2:**
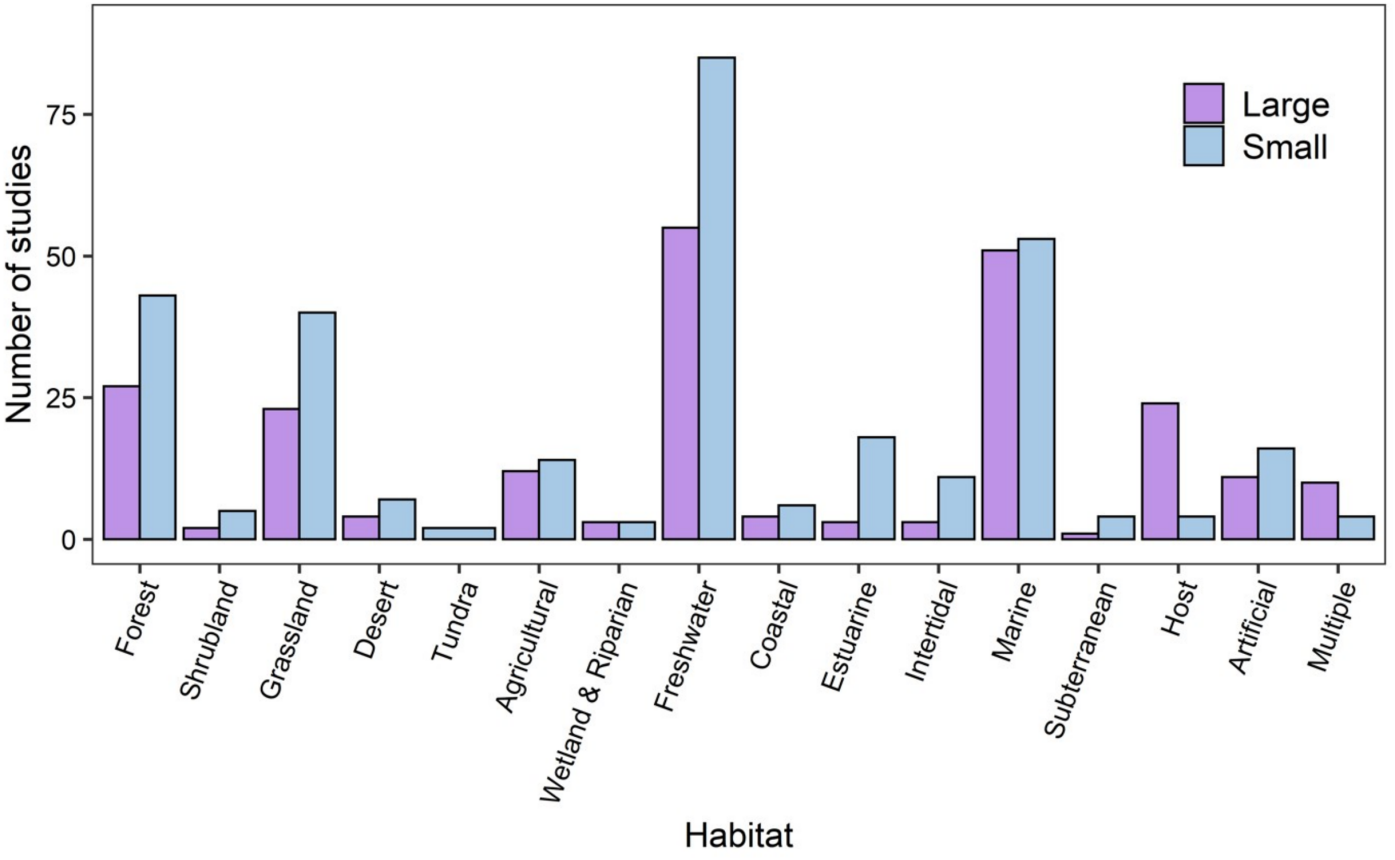
The numbers of studies that were conducted at large and small spatial scales across habitat categories. Small spatial scale (blue bars) studies were considered to be those carried out at micro, point sample, and local scales, while large spatial scale studies (purple bars) were those at regional, continental, or global scales. Marine studies include those in the open ocean in contrast to coastal (sandy coasts), intertidal (rocky shore) and estuarine.

Studies spanned a broad range of habitat types, taxa, spatial and temporal scales. A similar number of studies were conducted at small spatial extents (at micro, point sample and local scales) compared to large spatial extents (regional, continental or global). Across the 16 habitat categories identified from the articles, and the most frequently-sampled (69%) habitats were freshwater (141 studies), marine (106 studies), forest (72 studies) and grassland (69 studies) habitats. At small spatial extents, there were more studies in forest, grassland, freshwater, estuarine, intertidal and artificial habitats (Fig. 2).

**Figure 3:**
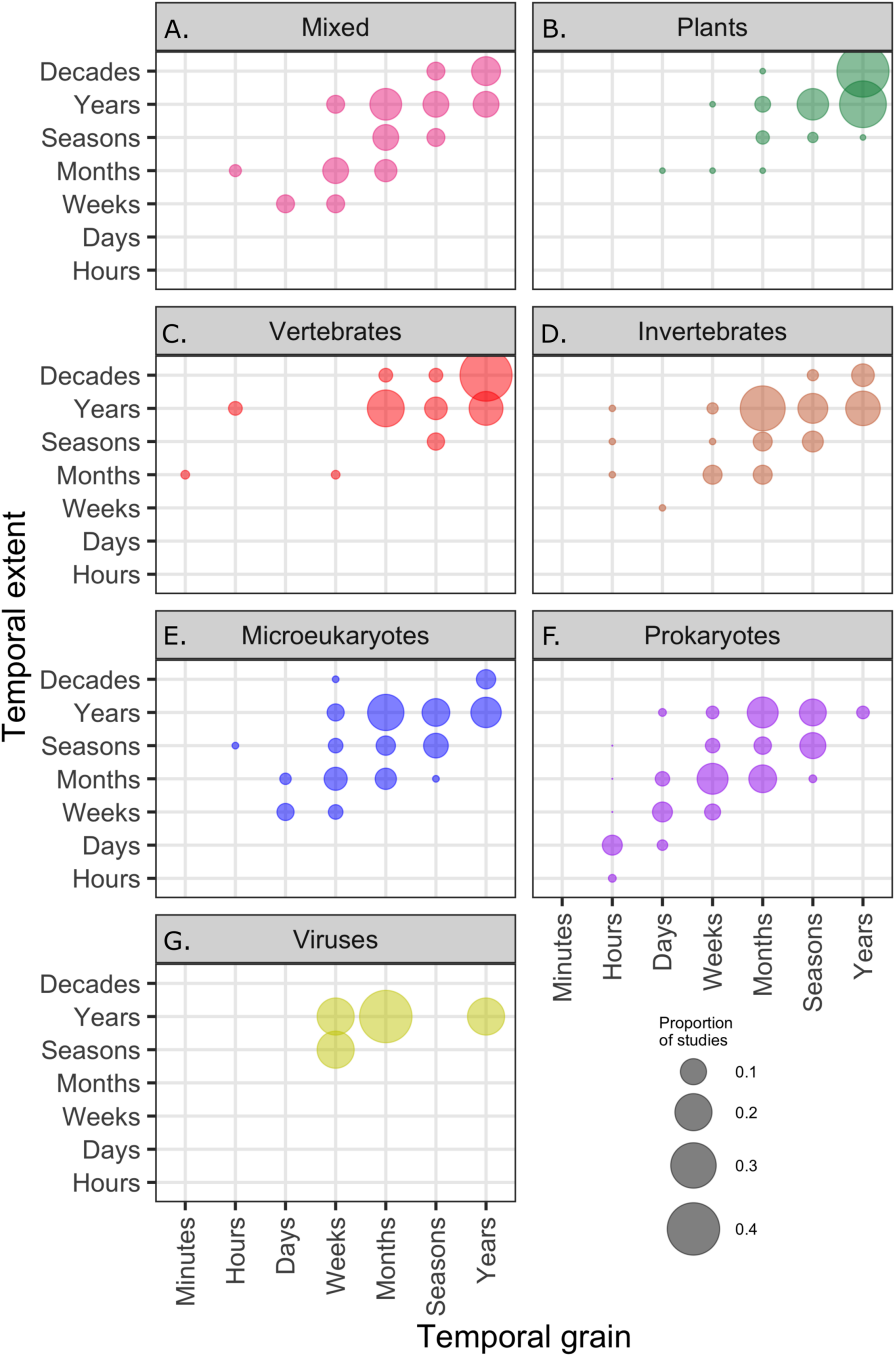
The proportion of studies of different taxa ((A–G) mixed taxa, plants, vertebrates, invertebrates, microeukaryotes, prokaryotes, and viruses) within categories defined by the temporal grain and extent; the proportions in each box sum to 1. Temporal grain is the minimum time between sampling events within the study and the temporal extent is the maximum time between sampling events within the study.

**Figure 4:**
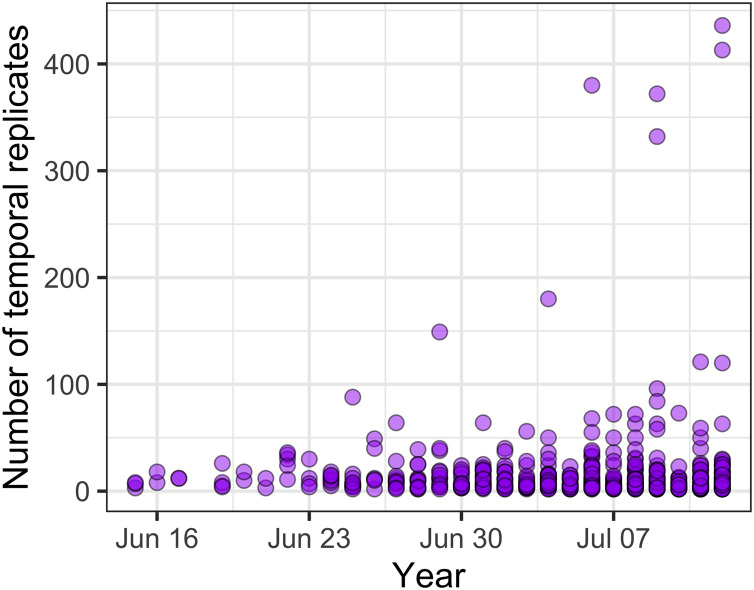
The number of temporal replicates in studies over time.

Across all communities, the most common temporal extent (i.e., the time between the collection of the first and last samples) of individual studies was years (46% of studies), and only a minority of studies, predominantly on plant and vertebrate communities extended over a period of one decade or longer (Fig. 3). Smaller organisms were often measured at finer temporal resolutions (grain) than larger organisms, but many studies on short-lived prokaryote and viral communities lasted more than one year (Fig. 3). The temporal grain of sampling generally was smaller for communities of microorganisms than for communities of vertebrate animals and plants, for which the most common grain for repeated sample collection was years rather than months or seasons (Fig. 3). Most time series (75%) had fewer than 15 temporal replicates (mean = 16.1, median = 7, mode = 4); a relatively small number of studies (12%) used 25 or more temporal replicates and this did not change substantially over time, although there was a small increase in the number of long time series (Fig. 4).

**Figure 5:**
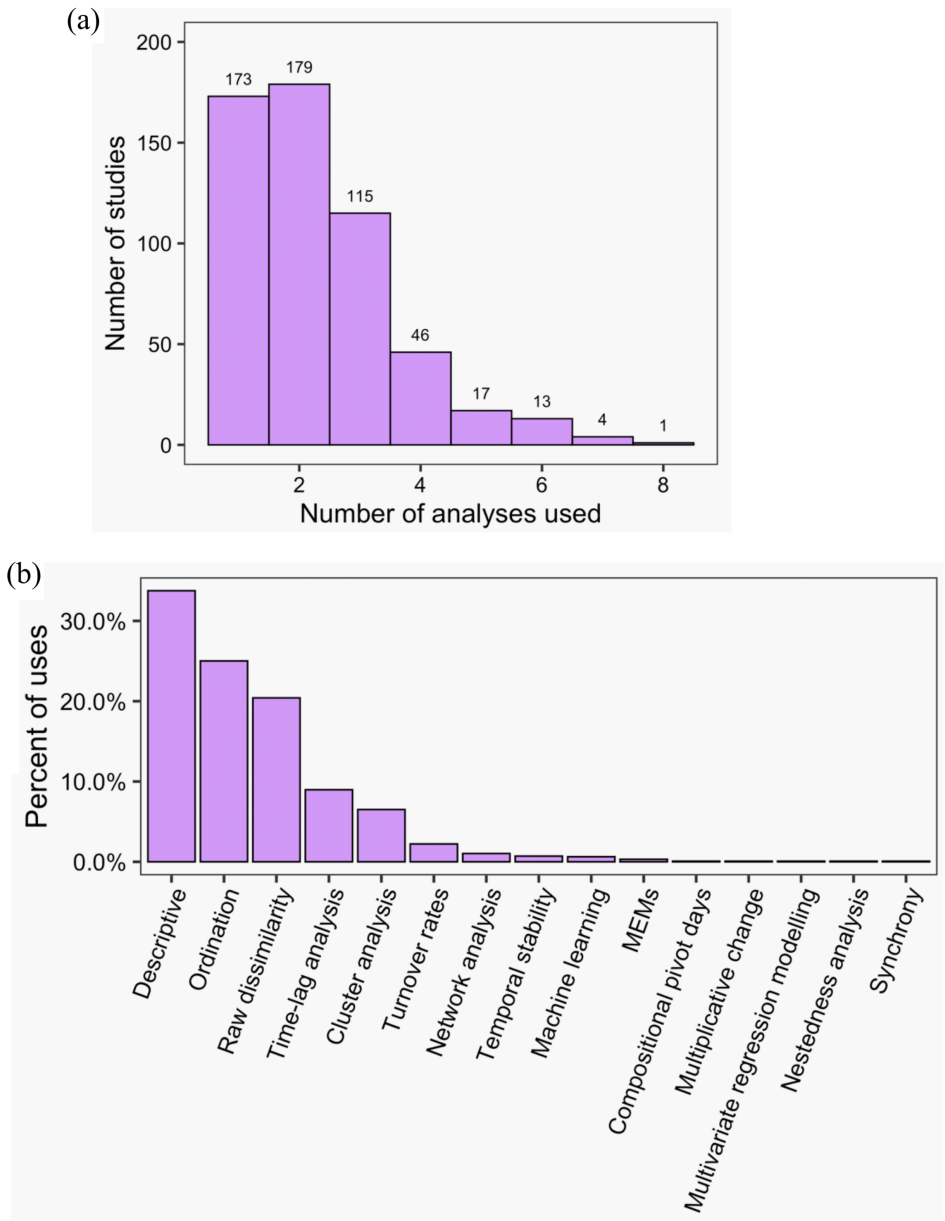
The frequency of use of different temporal community dynamics analysis methods across all reviewed studies. Specifically: (A) the number of different analyses applied in each study; (B) the percent of uses of each analysis type across all studies and times. MEMs refers to Moran Eigenvector Maps; see Table 1 for a brief explanation of each method.

**Figure 6:**
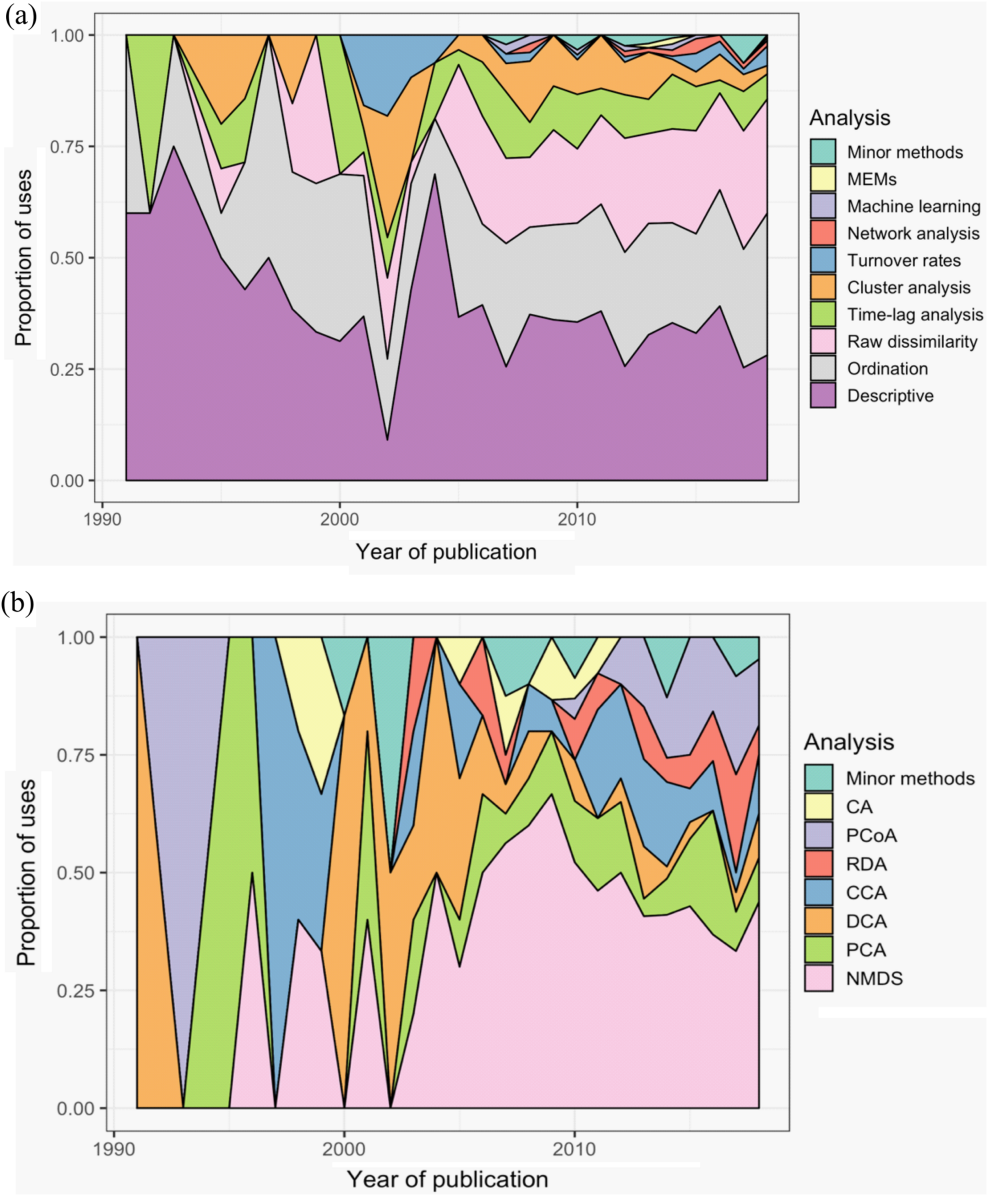
Trends in the use of different methods for the analysis of temporal community dynamics datasets, from 1990 to the end of 2018. (A) The proportion of uses of 10 categories of analysis methods across all sampled publications; ‘Minor methods’ include compositional pivot days, multiplicative change, multivariate regression modelling, nestedness analysis, synchrony, and temporal stability. (B) The popularity of the different ordination methods used across the 28 years. CA, correspondence analysis; PCoA, principal co-ordinates analysis; RDA, redundancy analysis; CCA, canonical CA; DCA, detrended CA; PCA, principal components analysis. ‘Minor methods’ are: distance-based RDA (dbRDA), detrended CCA (DCCA), partial CCA (pCCA), pRDA, Procrustes, RA and multiple co-inertia analysis. Additional details of each analysis method are provided in Table 1.

Across all studies, between one and eight methods were used to analyse community dynamics (Fig. 5A); 68% of studies (*n* = 375) applied more than one analysis method and 36% of studies (*n* = 196) used three or more methods. ‘Descriptive’ methods (i.e., visual, non-statistical comparisons) were used most widely (34% of all applied methods and used in 78% of all studies), with ordination (25% and 57%), raw dissimilarity (20% and 47%), time-lag analysis (9% and 21%), and cluster analysis (7% and 15%) comprising the next most common types of methods; the remaining 10 methods were used infrequently (Fig. 5). Although approaches to analysing community dynamics have begun to incorporate more computationally complex methods, descriptive and ordination methods have been consistently used since 1990, along with clustering and time-lag analyses (Fig. 6A). Since 2005, the use of raw dissimilarity methods has increased considerably. Ordination, clustering and time-lag analyses are also based on dissimilarity values, further illustrating the dominance of dissimilarity-based methods in community analysis. In the last 15 years, a steady stream of alternative temporal methods have been applied such as ‘compositional pivot days’ and synchrony, albeit with poor uptake across the wider literature. These methods often are ‘one-off’ analyses that have been applied only a handful of times (Fig. 6A). Preferred ordination methods changed from detrended correspondence analysis (DCA) and principal components analysis (PCA) in the early 1990s, to canonical correspondence analysis (CCA) in the late 1990s, and, since the early 2000s, to non-metric multidimensional scaling (Fig. 6B).

**Figure 7:**
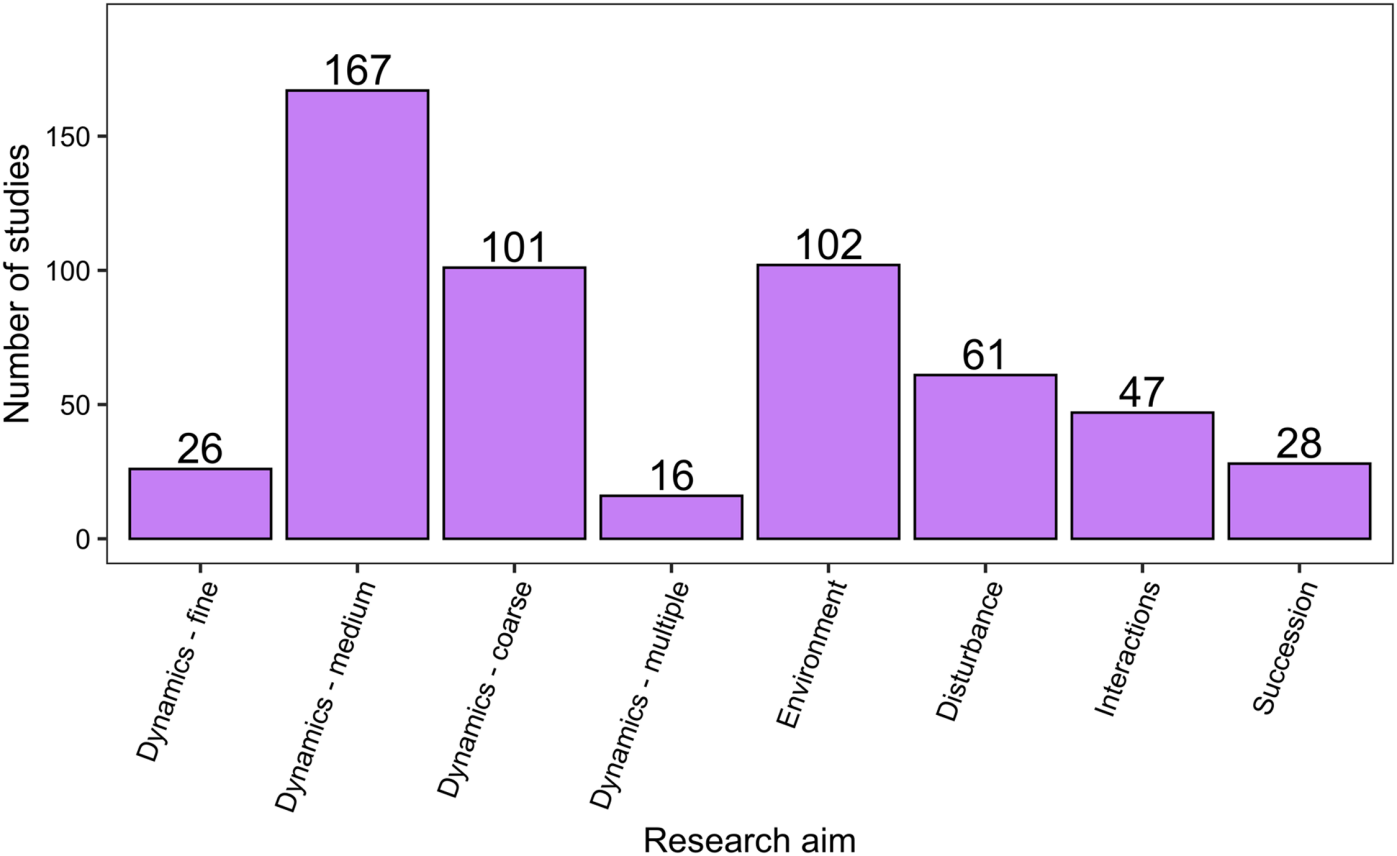
The distribution of studies across the different research aims. Research aims varied from understanding compositional dynamics at fine, medium and coarse temporal scales, to investigating the effect on species composition over time of environmental conditions, ecological disturbance, species interactions and understanding successional change in communities. Scales of temporal dynamics were classified as fine (days), medium (weeks, months, or seasons), and coarse (annual, years, or decades).

**Figure 8:**
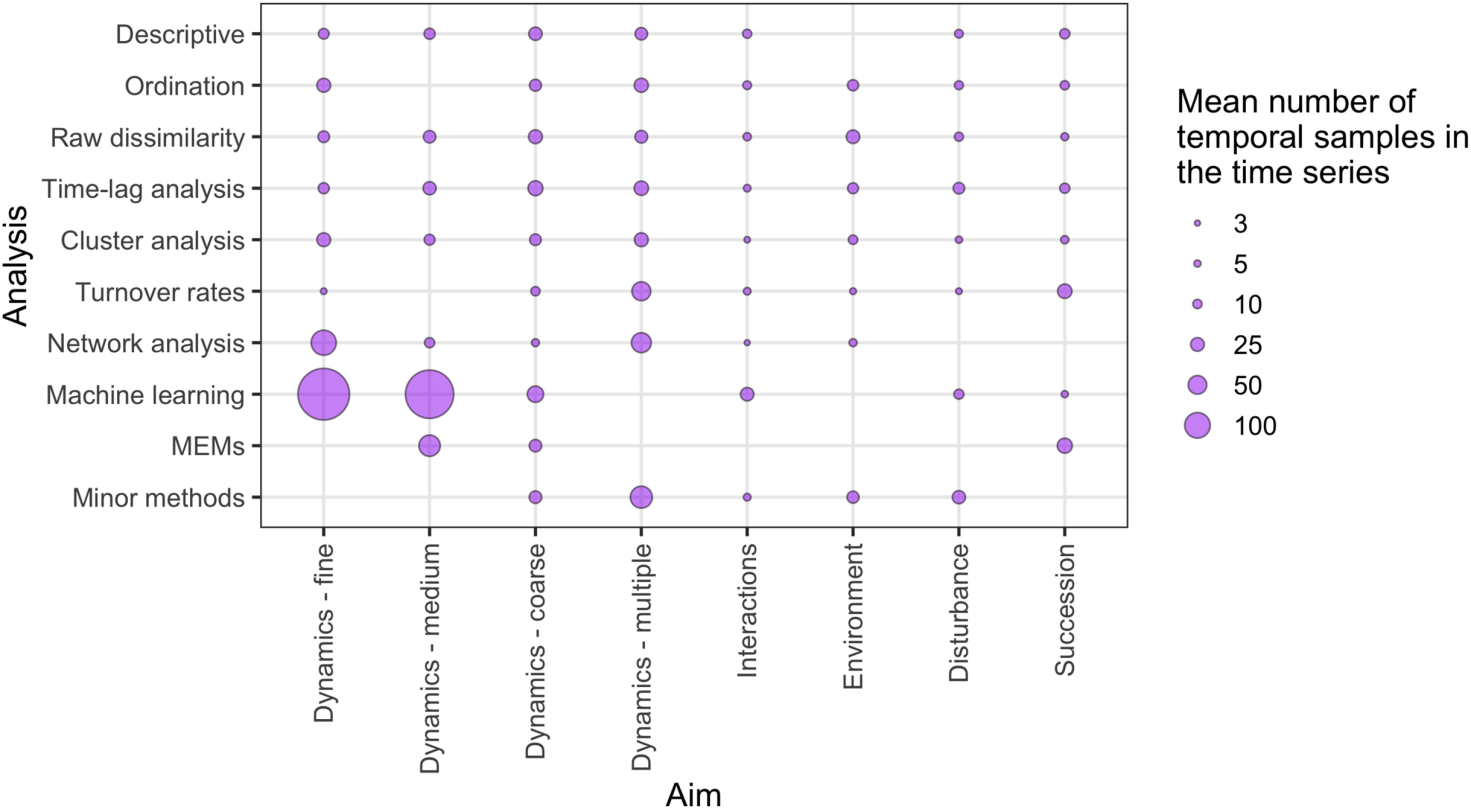
The relative distribution of the mean number of independent temporal samples across different research aims and temporal analysis methods. ‘Minor methods’ include compositional pivot days, multiplicative change, multivariate regression modelling, nestedness analysis, synchrony and temporal stability (see Table 1 for explanations of methods). Research aims vary from understanding compositional dynamics at fine, medium and coarse temporal scales, to investigating the effect on species composition over time of species interactions, environmental conditions, and ecological disturbance, to understanding successional change in communities.

There was a wide range of motivations for analysing community dynamics; most studies aimed to characterise temporal changes in community composition rather than exploring environmental effects or studying the effects of biotic processes or interactions (Fig. 7). Of the 562 studies, 406 were observational studies, 109 were field experiments and 47 were laboratory experiments. Research aims addressed community dynamics (*n* = 316), environmental effects (*n* = 107), disturbance and/or succession (*n* = 92) and species interactions (*n* = 47). For the vast majority of research aims and analysis types, community dynamics analyses were based on fewer than 25 temporal samples, with many based on ten or fewer samples (Fig. 8). Some complex methods require larger numbers of temporal replicates and may explain why they were seldom used. For example, Moran’s Eigenvector Maps, which is useful for detecting complex temporal patterns, was used by only five studies with relatively high temporal replicates: 20 (Kampichler et al., 2014), 31 (Matabos et al., 2014), 72 (Caruso et al., 2013) and 63 (Brasil et al., 2018).

## Discussion

We confirm that most community dynamics datasets are of short duration and include fewer than 15 temporal observations and commonly fewer than ten (the median temporal replication across all studies was seven observations). This is consistent with a previous review of experimental field studies (Vaughn & Young, 2010) and a general review of the spatial and temporal domains being studied by ecologists over the period of 2004–2014 (Estes et al., 2018). There was no relationship between the analysis methods selected and study aim. However, the relatively short temporal extent and grain of most studies conducted to date did appear to have impacted the methods used to understand community dynamics. For example, of the few studies that used MEMs, machine learning methods and multivariate regression modelling, most used more than 25 observations taken through time (median number of temporal samples = 31, mean number of temporal samples = 90.9 ± 143 S.D.). Thus, despite the recent rise of sophisticated statistical methods for temporal hypothesis testing in community ecology (e.g., Tonkin et al., 2017; Avolio et al., 2019; Legendre, 2019), the relative scarcity of long-term community datasets is likely to be limiting the wider use of these more data-intensive, strong inference methods. However, not all new statistical methods require long time series (e.g., De Cáceres et al., 2019).

The ecological literature applying multivariate methods of temporal community dynamics literature is large and diverse, covering a multitude of taxa, habitats, and research questions. Datasets collected encompass all spatial and temporal scales. There is little consistency across taxa, habitats or research questions in the analysis methods that have been applied; however, the majority of studies were focussed on primarily detecting patterns in temporal dynamics and, unsurprisingly, smaller, short-lived organisms were more likely to be studied at finer temporal scales than longer-lived organisms. Our understanding of patterns and drivers of changes of biological communities would benefit by having more longer-term studies (Kuebbing et al., 2018) conducted at finer temporal scales across the full range of taxonomic and habitat diversity. For instance, there is evidence that longer datasets enable the detection of non-linear dynamics (Giron-Nava et al., 2017). However, although permanent monitoring datasets, such as LTER sites, are generating longer datasets over time, such long-term studies are difficult to fund and support and there are recent suggestions that investment in such studies is declining (e.g., Lindenmayer et al., 2015; Vucetich, Nelson & Bruskotter, 2020). There is a pressing need for more analyses of long-term datasets because of their demonstrable value (Kominoski, Gaiser & Baer, 2018) if we are to understand and anticipate how community shifts influence, and are influenced by acute, seasonal and long-term climate change, for example.

Our understanding of temporal dynamics in biological communities and the methods available to investigate them have diversified over the past 29 years, ostensibly because of increases in computational power and in dataset size. A multitude of different data visualisation and statistical methods are available for understanding community change, many of which offer different sets of advantages and disadvantages. Descriptive methods that only visualise trends in the data, such as bar graphs or tables, and ordination remain the most commonly used analyses, regardless of the research aim. About one third of studies (*n* = 183) used only descriptive methods to analyse compositional community dynamics. However, restricting analyses to interpretation of descriptive and visual patterns limits the ability of researchers to either quantify or predict future ecological data trends. Indeed, it was clear that many researchers recognised the limitations of these descriptive analyses; 28% of studies used a descriptive method in combination with at least one other analytical method, which would enhance the ability to understand the complexities of any observed temporal patterns. Given the complexities inherent to temporal community dynamics datasets, we suggest that this type of multi-analysis approach better-enables researchers to reveal nuances of temporal change in their datasets (e.g., Ovaskainen et al., 2017b).

The increased use of methods based on raw compositional dissimilarities since the early 2000s may be because, like ordination and descriptive methods, these techniques work with community time series of any length. Methods based on raw community dissimilarity scores are flexible and can be used to analyse spatial and temporal variation simultaneously. For example, Collins (2000) used pairwise site-time sample dissimilarities to confirm directional (or ‘unstable’) change in plant species composition had occurred irrespective of fire history due to stochastic dynamics among rare satellite species. This ability to analyse spatiotemporal patterns in small datasets is important because spatial variability, if well-defined and understood, can aid the interpretability of temporal community dynamics (Collins et al., 2018).

Our review confirms the need for more long-term studies in ecology (Yang, 2020) and the application of multiple analytical approaches to increase understanding of the complexities of temporal community change. Researchers investigating both spatial and temporal dynamics are likely to benefit from using methods in addition to simple descriptive analyses and ordinations. For instance, methods based on raw community dissimilarities can be used to address relatively complex spatiotemporal questions using datasets of any size. With the growing array of traditional and newer methods available for temporal community dynamics analysis, additional guidance is needed for researchers to select the most appropriate methods for their research question and dataset (Buckley et al., 2021).

## Supplemental Information

10.7717/peerj.11250/supp-1 Supplemental Information 1 The dataset assembled for the literature review.

## Additional Information and Declarations

### Data Availability

The following information was supplied regarding data availability: The raw review data compiled for this article are available in the Supplemental File.
